# Perspectives of Medicare Advantage Plan Representatives on Addressing Social Determinants of Health in Response to the CHRONIC Care Act

**DOI:** 10.1001/jamanetworkopen.2019.6923

**Published:** 2019-07-12

**Authors:** Kali S. Thomas, Shayla N. M. Durfey, Emily A. Gadbois, David J. Meyers, Joan F. Brazier, Ellen M. McCreedy, Shekinah Fashaw, Terrie Wetle

**Affiliations:** 1 Providence VA Medical Center, Providence, Rhode Island; 2 Center for Gerontology and Healthcare Research, Brown University School of Public Health, Providence, Rhode Island; 3 Warren Alpert Medical School, Brown University, Providence, Rhode Island; 4 Department of Health Services, Policy, and Practice, Brown University School of Public Health, Providence, Rhode Island

## Abstract

**Importance:**

The passage of the Creating High-Quality Results and Outcomes Necessary to Improve Chronic (CHRONIC) Care Act in 2018 allows Medicare Advantage (MA) plans, which enroll more than one-third of Medicare beneficiaries, greater flexibility to address members’ social determinants of health (SDOH) through supplemental benefits.

**Objective:**

To understand MA plan representatives’ perspectives on the importance of addressing members’ SDOH and their responses to the passage of the CHRONIC Care Act.

**Design, Setting, and Participants:**

This semistructured qualitative interview study conducted via telephone from July 6, 2018, to November 7, 2018, included participants from 17 MA plans that collectively enrolled more than 13 million MA members (>65% of the total MA market). Data analysis was conducted from September 18, 2018, to December 13, 2018.

**Main Outcomes and Measures:**

Audio-recorded interviews were transcribed and then analyzed using a modified content analysis approach to identify major themes and subthemes.

**Results:**

Thirty-eight participants representing 17 MA plans varying in region, star rating, and size were interviewed. Analysis of interviews revealed 3 key themes. The first theme was that participants increasingly recognize the value of addressing members’ SDOH. The second theme was that participants had different perspectives on whether MA plans should directly address SDOH and how to do so. While some reported that they were taking advantage of the increased flexibility provided by the CHRONIC Care Act to design new benefits or partner with community-based organizations, others indicated that it was outside of their purview to directly address members’ SDOH. The third theme was that participants described complex decision-making around how to provide supplemental benefits, including a need for evidence, return on investment, strong community partnerships, and guidance from the US Centers for Medicare & Medicaid Services.

**Conclusions and Relevance:**

These findings suggest that the changes in MA plans’ benefit packages in response to the CHRONIC Care Act and their efforts to address SDOH will vary. Therefore, it is likely that MA enrollees will be differentially affected by the implementation of the CHRONIC Care Act.

## Key Points

**Question:**

How are Medicare Advantage (MA) plan representatives responding to new flexibility granted by the Creating High-Quality Results and Outcomes Necessary to Improve Chronic Care Act to address members’ social determinants of health through supplemental benefits?

**Findings:**

This qualitative study with participants from 17 MA plans (representing >65% of the MA market) revealed that addressing members’ social determinants of health was important but reported 2 distinct approaches: creating supplemental benefits or supporting community-based organizations. Participants described complex decision-making concerning how to provide supplemental benefits, including a need for evidence, return on investment, strong community partnerships, and US Centers for Medicare & Medicaid Services guidance.

**Meaning:**

Enrollees in MA may have differential access to supplemental benefit offerings that address social determinants of health.

## Teaser

This qualitative study examines the perspectives of representatives of US Medicare Advantage plans on how their organizations can enact programs to promote social determinants of health in light of expansions offered under the Creating High-Quality Results and Outcomes Necessary to Improve Chronic (CHRONIC) Care Act.

## Introduction

Social determinants of health (SDOH) may have a larger impact on individuals’ health and well-being than medical care.^1^ It is estimated that where people live, work, and socialize determines as much as 60% of their health, whereas formal medical care accounts for just 10%.^2^ Older adults who are socially connected, food secure, and financially stable and have easy access to transportation are healthier^3,4,5^ and have lower health care utilization.^6,7,8,9^ Thus, the health care system has an increasing interest in addressing SDOH as a way to decrease costs and improve health. This has been reflected in several recent US Centers for Medicare & Medicaid Services (CMS) initiatives.^10,11,12^

Medicare Advantage (MA), which enrolls more than one-third of all Medicare beneficiaries,^13^ has the potential to influence health by offering services beyond medical care. In MA, private health insurance plans are paid on a capitated basis to cover members’ health care needs. Thus, MA plans have an incentive to address their members’ SDOH to reduce unnecessary health care utilization and contain costs. Medicare Advantage plans have historically provided supplemental benefits that are not covered under traditional Medicare (eg, dental benefits, eyeglasses, gym memberships, meals after hospital discharge, medical transportation) to members. Until recently, regulations required that MA plans’ supplemental benefits be primarily health related and offered to all members. However, the Creating High-Quality Results and Outcomes Necessary to Improve Chronic (CHRONIC) Care Act of 2017, passed as part of the Bipartisan Budget Act of 2018^14^ (effective 2020), allows greater flexibility in what benefits may be offered.

The CHRONIC Care Act was passed by Congress in a bipartisan effort to impel the Medicare program to be more responsive to the needs of beneficiaries with chronic illnesses.^15^ There were several key changes under the act. First, plans were given the opportunity to design supplemental benefits that “have a reasonable expectation of improving or maintaining the health or overall function”^16^ of chronically ill members and target these benefits to members at greatest risk. These benefits could include diverse services, such as meal delivery, cooking classes, home modifications to assist with mobility, personal care services, and many others.^17,18^ There is no requirement that plans offer these benefits, and as a result, there may be variation in how plans respond to these changes. In addition to expanding supplemental benefits, the CHRONIC Care Act waives the requirement that all members be offered uniform access to benefits, enabling more specific targeting of services to members who may benefit the most.

To our knowledge, very little is known about MA plans’ interest in taking advantage of the new flexibility for benefit design that the CHRONIC Care Act offers to address their members’ SDOH or whether barriers exist to providing these services as part of their plans’ benefit structure. Prior to the CHRONIC Care Act, some MA plans announced efforts to address SDOH. For example, Humana’s Bold Goal^19^ initiative includes several programs addressing healthy eating and social isolation. However, this and other MA plans’ initiatives from before the CHRONIC Care Act to address SDOH were funded through case management or with administrative dollars, as opposed to a formal benefit for members. As such, these efforts may have been somewhat limited. The objective of this article is to understand the perspectives of MA plan representatives on the importance of addressing members’ SDOH, the challenges they face in doing so, and their responses to the passage of the CHRONIC Care Act.

## Methods

We conducted semistructured interviews with participants from MA contracts (referred to as *plans*). Plans were identified using purposive and snowball sampling approaches. We first recruited representatives from plans that we knew were knowledgeable about many of the topics of interest. Representatives then were either recruited or connected us with other appropriate representatives within their plans. Then, we asked participants for recommendations of other potential plans and participants. We purposefully recruited plans of varying size, quality, and geographic location.

We designed semistructured interviews to understand MA plan representatives’ perspectives on addressing members’ SDOH and responses to the CHRONIC Care Act. We drafted the interview guide and piloted it with 1 MA plan, making subsequent revisions to questions that were difficult to interpret or failed to elicit intended responses. We emailed the interview guide to participants in advance to confirm that relevant, knowledgeable plan staff were included in the interview. One of us (E.A.G.) conducted the interviews via telephone from July 6, 2018, to November 7, 2018. Interviews lasted approximately 1 hour and were recorded with participants’ consent. This project was deemed exempt from institutional review board approval by the Brown University Institutional Review Board because it was not considered human subjects research. We continued to recruit plans of various characteristics until data saturation was achieved,^20^ meaning that no new information or themes were observed through additional interviews. This study is reported following the Consolidated Criteria for Reporting Qualitative Research (COREQ) reporting guideline.

Transcripts were analyzed using a modified content analysis approach to identify overarching concepts and themes.^21,22,23,24^ We first developed a preliminary coding scheme based on the questions in our protocol, then used an iterative process to add codes and refine code definitions. The resulting coding scheme reflected both the a priori codes and codes for unanticipated content.

At least 2 of us (E.A.G., J.F.B., E.M.M., S.F., and T.W.) coded each transcript. For each transcript, we discussed preliminary patterns and reconciled our interpretations. We kept a comprehensive audit trail that recorded ongoing team decisions, including selection and definitions of codes and discussion of emerging themes.^22,25,26,27,28^ Coded data were entered into the qualitative software package NVivo version 12 (QSR International) for data management. For anonymity of the participating MA plans, we identify organizations by numbers assigned for this study. Data analysis was conducted from September 18, 2018, to December 13, 2018. For additional details, see the eAppendix in the Supplement.

## Results

Interviews were conducted with 1 to 6 participants per plan for a total of 38 participants from 17 MA plans. Participant positions included presidents or chief executive officers, chief medical officers, government affairs officers, chief legal officers, directors of health policy, and various vice presidential roles. Participants had been in their positions for 1 to 30 years. Represented plans were both national and regional in scope, had a range of publicly reported CMS quality star ratings^29^ (scale, 1-5, with 5 stars indicating the highest quality), and enrolled from fewer than 50 000 members to more than 3 million members. Participating plans enrolled more than 13 million MA members in 2018 (>65% of the total MA market) (Table 1).

**Table 1.** Descriptive Characteristics of Medicare Advantage Plans Represented by Study Participants^a^

Organization	Plan Scope	Star Rating^b^	Enrollment, No.^c^
1	Regional	<3	<50 000
2	National	3-4	≥3 Million
3	Regional	3-4	100 000-250 000
4	Regional	3-4	50 000-100 000
5	National	3-4	<50 000
6	National	3-4	>3 Million
7	Regional	>4	50 000-100 000
8	Regional	>4	50 000-100 000
9	Regional	New^d^	<50 000
10	Regional	>4	100 000-250 000
11	Regional	>4	100 000-250 000
12	Regional	>4	100 000-250 000
13	Regional	>4	<50 000
14	National	3-4	250 000-500 000
15	Regional	>4	50 000-100 000
16	National	3-4	250 000-500 000
17	Regional	>4	100 000-250 000

^a^ Plan characteristics have been rounded and organizations are identified by numbers assigned for this study to protect plan anonymity.

^b^ Star ratings were identified based on 2018 publicly reported US Centers for Medicare & Medicaid Services star rating data.

^c^ Plan enrollment is the total enrollment from all plans owned by the organization in 2018 from publicly available contract enrollment files.

^d^ New plans are not eligible for a star rating until they have been active for at least 3 years.

Our analysis found that representatives of MA plans believe addressing SDOH is important to improving the health of members and the health care delivery system. However, perspectives on the role of MA plans in addressing SDOH varied; while some participants reported that their plans are taking advantage of the increased flexibility in benefit design provided by the CHRONIC Care Act, others believed it was outside the purview of their plan to directly address members’ SDOH and were instead looking to collaborate with or refer to community-based organizations (CBOs). Participants reported their decision-making process for evaluating the many, sometimes conflicting, pressures they were weighing in responding to the CHRONIC Care Act and addressing SDOH, and some participants noted being cautious about moving forward in this area without clearer guidance from CMS. In this study, we discuss each of these themes and include illustrative quotes from interview participants.

### Theme 1: Addressing SDOH Is Important to Improving the Health of Members and Enhancing the Overall Health Care Delivery System

Participants from MA plans recognized the importance of addressing SDOH for improving both member health and the larger health care system. Participants highlighted how addressing SDOH enables members to remain in the community and reduces health care costs. Participants also described SDOH as an increasingly discussed topic in MA, especially in response to legislative and policy changes like the CHRONIC Care Act. Representative quotes are presented in Table 2.

**Table 2.** Key Concepts, Representative Quotes, and Medicare Advantage Plans’ Characteristics for Theme 1

Key Concepts	Representative Quote	Medicare Advantage Plan Organization^a^	Organizational Characteristics
Enabling members to stay in the community and reduce health care costs	“We really try to keep people in the community as much as possible, and that means providing everything from transportation to home-delivered meals (obviously when it's appropriate), to durable medical equipment and things that will make it possible to keep people in their home....We feel pretty strongly that if we are able to maintain people in the community and address their social determinants that their health care costs go down. So it’s a pretty high priority.”	13	Regional, >4 stars, <50 000 members
Increased focus on SDOH	“[Social determinants of health] certainly [are] becoming much more of an area of focus for us. It’s certainly a bigger blip on our radar screen than it had been in the past. You can’t go to any type of event where health care improvement is being discussed in the state or a national event or whatever, or even some of our own internal meetings, and the phrase *social determinants of health* comes up.... Because it is clear that it plays a big role in people’s health.”	7	Regional, >4 stars, 50 000-100 000 members
	“This space is evolving quickly, and I think between the CHRONIC Care Act and changes that CMS is making and then just broader evolution in the health insurance sector in general with this big shift and focus of social determinants of health. I think there’s going to be a lot happening in this space. And there’s a lot of great potential here.”	2	National, 3-4 stars, >3 million members
Holistic approach exploring social isolation, housing, and caregiver supports	“We look at people in a holistic manner, and so there’s only so many things we can do through the benefit structure, so we can have transportation. We can have those discharged meals, but there’s other issues, such as social isolation and even caregiver support for seniors.... Now that CMS is kind of championing more flexibility, those are things that we’re able to explore further...things like social isolation, housing.... It’s not just about a PCP copayment or inpatient stay benefit. It’s more about the other things, the ancillary things that people have to deal with on a day-to-day basis that they can’t get help through their health plan, and we’re trying to find ways to help them in that respect.... So one example is we looked at the caregiver support last year, and myself and our community outreach manager, we went and met with an entrepreneur. She has sort of a phone support staff. What they do is they actually talk to caregivers. They try to coach caregivers. ‘Hey, make sure you take time for yourself. What you’re doing is very noble for your family member, but you got to make sure to take time for yourself. Take a walk, do yoga, those sorts of things.’ You’re seeing more and more of that across Medicare. It’s not just us. The Medicare Advantage plans are looking at those types of things.... If we were going to do new and exciting things, social services [or] social determinants [are] kind of where we can make a dent and an impact.”	10	Regional, >4 stars, 100 000-250 000 members
Addressing food insecurity and social isolation	“We have been interested in looking at new and innovative opportunities to address social determinants, but today, our primary focus has been around 2 social determinants, one being food insecurity, the other one being inadequate social support. So it presents through social isolation and/or loneliness. But we’re always interested in new opportunities to extend that impact, especially with points to improve the health of members.”	6	National, 3-4 stars, >3 million members
Services being considered: transportation, home-delivered meals, and housing	“The idea of providing transportation to office visits or the idea of alternate services that previously had not been reimbursed, such as home delivery of meals, even things like housing for patients—those are things that had previously not been considered medically necessary services, but we are taking a very serious look now at all of those things and more to try to find ways to address those particular issues.”	7	Regional, >4 stars, 50 000-100 000 members

Abbreviations: CHRONIC, Creating High-Quality Results and Outcomes Necessary to Improve Chronic; CMS, US Centers for Medicare & Medicaid Services; PCP, primary care physician; SDOH, social determinants of health.

^a^ Organizations are identified by number for anonymity.

Participants from MA plans discussed multiple aspects of SDOH that are associated with their members’ health, especially in regards to keeping people safely in their homes and communities. These participants highlighted their desire to further efforts to provide meals and transportation beyond the previously allowed posthospitalization meals and medical transportation and also noted that legislative changes may allow them to view their members in a more holistic manner (organization 10) and attempt to address other important issues, such as social isolation, housing, and caregiver support. Representative quotes are presented in Table 2.

### Theme 2: Perspectives on Whether MA Plans Should Directly Address SDOH and Their Methods for Doing So Varied

Participants from MA plans consistently discussed the importance of members’ social and lifestyle factors to their overall health and health care utilization. However, participants offered divergent perspectives on whether plans should directly address SDOH. While some were excited about offering new benefits or expanding existing services to address SDOH, others questioned the appropriateness of expanding their purview to include nonmedical care. Given these differences in perspectives, 2 distinct methods of providing SDOH-focused services emerged: (1) offering a supplemental benefit or (2) supporting CBOs and referring members to these organizations to receive needed services.

#### Plans Seek to Address SDOH Through Formal Benefit Design

With the increased flexibility for benefit design made possible with the passage of the CHRONIC Care Act, some participants discussed their motivation to introduce a new benefit to address members’ SDOH and expand the services that they had already been providing. Participants also noted the value of the CHRONIC Care Act in allowing plans to design benefits that are targeted for specific subgroups of members. Some participants mentioned that offering a new supplemental benefit to address SDOH is a way to meet consumers’ needs and expectations; therefore, this could allow them to be more competitive in the marketplace and increase their membership. One participant believed adding a new benefit to address members’ SDOH was “the right thing to do,” particularly if it “makes sense from a quality and cost and member experience” perspective (organization 4). Representative quotes are presented in Table 3.

**Table 3.** Key Concepts, Representative Quotes, and Medicare Advantage (MA) Plans’ Characteristics for Theme 2

Key Concepts	Representative Quote	MA Plan Organization^a^	Organizational Characteristics
**Plans Seek to Address SDOH Through Formal Benefit Design**			
Value of including benefits to address SDOH; potential expansion	“We very much believe in the importance of offering social supports to our members. Our enhanced benefits structure is actually the most generous benefit structure in the state.... We do offer meals postdischarge for our Medicare Advantage plan. We do offer access to free health education classes that focus on chronic disease and also on exercise, and we offer free transportation to and from those classes. We have free gym fitness memberships. We offer [a weight-loss program], which actually has really nice utilization by the numbers, chiropractic care, vision and dental enhanced benefits, and personal emergency response system devices...I mean, we have many of these programs already in place. And when CMS recognizes it, when our own state recognizes it, it means the conversation is easier to have. So, it’s easier to pull people in. It’s also becoming a bit competitive, which frankly is wonderful. If all the health plans are trying to help people with social determinants, that’s a good thing.”	15	Regional, >4 stars, 50 000-100 000 members
Ability to develop benefits to specific populations	“One challenge that the MA organizations have had for years has been that even if we have identified specific populations that have the need for specific programs, the requirement has always been that we provide them regardless of the need to everybody evenly. That is the new opportunity that we have and why we have started to stratify the population into a meaningful crux within a benefit option.... In future years, we’ll have the ability to develop programs and benefits that are more targeted than maybe has been the case in the past.”	1	Regional, <3 stars, <50 000 members
Offering a benefit to meet consumers’ expectations and to provide a value differentiation in competitive market	“We’ve always heard about gym memberships being included in an MA product, but I think it’s going a step further now. Now, it’s getting into, ‘Is there a nutritionist? Do you have somebody that’s actually willing to go to the grocery store with me? Could you sign me up for a cooking class?’ Things that are just above and beyond what we’ve ever seen before, and it’s being produced from that consumer block. So those expectations are evolving, rightfully so, as they all have indirect relationships to utilization and cost management. We all see it as being in the appropriate bucket, but it’s great when you see the actual beneficiaries expecting it, because it means that they’re going to engage [in] that. We’re not prescribing it as much as we’re meeting a need of the market....Well, fortunately, we have great relationships in our markets, and I think our MA business, from top down, just ensures that they’re constantly in the market, listening...we have to ensure that we’re all on the same page, and those conversations lead to those new types of expectations, that we’re trying to create a value differentiator for our plan.”	11	Regional, >4 stars, 100 000-250 000 members
Offering a benefit to increase membership	“I’m looking to grow my membership. We’re a for-profit company, so the other thing that I’m looking at is what are going to be the things that are [going to] resonate out in marketplace that people want to see and want to have.”	14	National, 3-4 stars, 250 000-500 000 members
Desire to offer a benefit because it is the right thing to do and controls costs	“I think we feel pretty strongly that it [offering a new benefit to address SDOH] is the right thing to do. Then on top of that, if you can align the right thing to do with the cost, then it’s a no-brainer. So what we want to do is to look at, right now, there’s still a lot of people that continue to get admitted to the hospital or go to the [emergency department], and it’s avoidable…. [Emergency departments] and hospitals are less-than-optimal places for older adults, and they [older adults] tend to lose function, get more confused, get infections, all those kinds of things in those kinds of settings. So, whatever we can do to provide treatment in place and proactive care and intervention makes sense from quality and cost and member experience. So that’s that whole triple aim, making sure that we’re doing that.”	4	Regional, 3-4 stars, 50 000-100 000 members
**Plans Seek to Address SDOH by Referring to and Supporting CBOs**			
Whose responsibility: MA plan or community?	“I think there are some important considerations as a health plan that we need to make as to what business is it of ours to engage in this kind of work?...We have not thought of ourselves as potentially the entity to solve those problems, but that doesn’t mean we may not innovate to that in the future. We’re very early in our strategy kind of decisions around how we want to continue to partner with community agencies and perhaps get into areas of business that, as a health plan, we have not been in before.... That is the conundrum I think we’re in, in terms of what degree do we really want to innovate and get into lines of business that really are best served by providers or communities at large. It’s a fundamental question I think that we’re still working through.”	12	Regional, >4 stars, 100 000-250 000 members
Addressing members’ needs is “a village effort”	“There’s a mosaic here, and we know that we aren’t yet able to cover all the pieces of this mosaic. If you have a piece that you can add, whether that’s education and support or something totally different, we’re completely open-minded to that. And in fact, we support anyone who is supporting us in enhancing community-based care because, ultimately, this is a village effort. I don’t think there’s going to be a day where the payer just steps in and saves everything and fixes everything.”	5	National, 3-4 stars, <50 000 members
Leveraging community resources to enable referrals	“Increasingly, we’re looking at care management programs that leverage community resources. We have a program that is kind of an aggregator of those services that is made available to our care managers that they can then bring to bear when they’re engaging our members and they find that they have needs that are kind of outside of our benefits. So that is our more community-based kind of person-centered multidisciplinary care management program. It’s becoming increasingly local like that, and it is focusing more and more on addressing those social determinants with community-based purposes.”	2	National, 3-4 stars, >3 million members
Investing directly in CBOs	“These community agencies have often really important long-term relationships with our members. That’s really something we want to enable and empower them to continue to do without necessarily having to rely on their health plan to do so. How do we better equip community agencies that surround us to provide the supports that are really critical to advancing health and overall well-being?... I know off the top of my head at this point in time, we’re investing close to a quarter of a million dollars in addressing social needs through community grants.... Our communities are best positioned to be able to address their own needs. They’re closest to it.”	12	Regional, >4 stars, 100 000-250 000 members
Plans cognizant of potential duplication of efforts	“Our philosophy as we kind of get into the social determinant space is that we want to serve as an anchor system to help our community partners be successful. We feel that we have a responsibility as a health care organization to do that, but, in the same sense, we don’t want to build these services out.... We don’t want to build our own fleet of cars and provide transportation and become a transportation vendor. We really want to develop programs that can help our community partners be sustainable.”	17	Regional, >4 stars, 100 000-250 000 members

Abbreviations: CBO, community-based organization; CMS, US Centers for Medicare & Medicaid Services; MA, Medicare Advantage; SDOH, social determinants of health.

^a^ Organizations are identified by number for anonymity.

#### Plans Seek to Address SDOH by Referring to and Supporting CBOs

Other participants questioned whether it was the plan’s responsibility to address members’ SDOH and proposed that these needs might be “best served by providers or communities at large” (organization 12). Some mentioned the importance of a collaborative effort by plans, community agencies, and other stakeholders to fully address members’ needs. These participants preferred to address members’ SDOH by referring to and supporting CBOs. One participant, recognizing the strength of organizations in the community, described developing a program that aggregates community resources and is used by care managers to identify services and make referrals for members. Others discussed investing directly in CBOs (eg, through community grants) to enable these organizations to provide needed services to members and help “community partners be sustainable” (organization 17). Participants highlighted being cognizant of not duplicating the efforts of CBOs that have existing expertise in addressing SDOH. Representative quotes are presented in Table 3.

### Theme 3: Participants Described Complex Decision-making Concerning How to Address Members’ SDOH Following the Passage of the CHRONIC Care Act

Participants described detailed decision-making efforts regarding how best to meet members’ social needs. For plans that are considering addressing SDOH through a formal benefit, 3 overarching topics emerged as critical to decision-making concerning providing SDOH-focused services: financial return on investment (ROI), capabilities of community agencies to partner with plans, and guidance from CMS.

#### Participants Described ROI and Evidence of Success as Central to Decision-making Concerning Which Services to Provide

One major factor for deciding how to address SDOH that participants mentioned concentrated on ROI and whether a new supplemental benefit would result in savings. Given that the CHRONIC Care Act newly allows plans to target benefits to certain populations, plan representatives are also considering the complexities concerning choosing which groups to target for these supplemental benefits. Thus, participants expressed a need for more evidence on which benefits or services would be most effective in improving health outcomes and reducing costs and on determining which populations to target. Some plans mentioned the need for evidence that adding a benefit to address members’ SDOH would improve their star ratings, an important measure of MA plan quality that results in bonuses or penalties for plans. One participant described efforts to build that evidence base through pilot programs to eventually transform effective interventions into benefits. In making decisions about what benefits to provide, participants also reported examining research articles and engaging with members about their needs using focus groups. Participants also reported that members switching plans, members’ many needs, and varying operating margins from county to county made developing an evidence base particularly complex for MA. Representative quotes are presented in Table 4.

**Table 4.** Key Concepts, Representative Quotes, and Medicare Advantage Plans’ Characteristics for Theme 3

Key Concepts	Representative Quote	Medicare Advantage Plan Organization^a^	Organizational Characteristics
**Participants Described ROI and Evidence of Success as Central to Decision-Making Concerning Which Services to Provide**			
ROI	“When you’re looking at supplemental benefits and other services through that sort of channel, we need to look at how much they cost, what the benefits are relative to other benefits. Because there’s a limited amount of funding contained within supplemental benefits, we can’t afford to pay for someone’s meals and rides every single day.... So I think even as we’re allowed to do more, we’re going to have to weigh what do consumers want, what kind of resources do we have available to fund these. And then, clinically..., where are we going to get the biggest bang for our buck to provide us maybe one of these services that sort of fall in the social determinants health bucket, but where it’s actually going to move the needle to help somebody that has a chronic condition.”	2	National, 3-4 stars, >3 million members
	“It’s all tied to ROI. So, does it make sense financially? Can we cover it within the premium? I mean, we’re capitated, and so we just can’t spend money without justifying it through savings in some way. So the impact of the nonmedical services has to be clear. If it isn’t clear, then we probably shouldn’t do it.”	15	Regional, >4 stars, 50 000-100 000 members
Determining which populations to target and with what benefits	“So, how our priorities have changed is, as soon as we finished our bids for 2019, we already kicked off a work group looking at what we’re going to do for 2020, which was probably substantially ahead of what we would have done in the past, to looking at where can we make an impact. Where could we do things that might be chronic disease–specific to help include the outcome for the lives of those individuals? ... Do you go out and provide air conditioners for folks who have COPD, to help remove the contaminants from the air if they’re living in very hot and humid climates?... Do you provide more meals to folks who are diabetic to get them on the right track if they’re newly diagnosed or people who need to be on a low sodium diet?... Or do you just get on a schedule of providing X number of meals to folks, period? And again, you can do it disease specific. You could do it to your overall population. You’d have to see how much money you have to spend to help keep them from being admitted to the hospital and, again, have healthier lives…. We look at how much of this population do we have, we look at our data and say, ‘Is this an area where we [have] a significant population that we could better impact their lives, better impact their outcomes? What are the issues that they’re struggling with or we can perceive that they’re struggling with?’ We do focus groups. We buy data. We also look at a lot of research articles whenever I want to put in a new benefit.... You can’t just do everything. So, we have to do the things that are [going to] provide us with the most impact, and that would be outcomewise.”	14	National, 3-4 stars, 250 000-500 000 members
Need for an evidence base and pilot studies	“We are definitely interested in social determinants of health.... We are restricted to social determinants that impact health and health outcomes.... There isn’t necessarily an evidence base yet that shows if you trust these things you’re going see a lower total cost of care. While we are endeavoring in the future when we can to incorporate these things into supplemental benefits, in the meantime, we are looking at ways of exploring other pile-ups and interventions to build that evidence base to really get those proof points to justify coverage in the benefits structure.... Without saying specifics, really the way we started this work was to look at which social determinants would have the biggest impact on health-related quality of life. So we’ve done some research that really led us down the path of focusing on food insecurity, social isolation, and then a few others, like transportation. Then what we’ve seen…as we’ve started down the path of doing pilots and different interventions, is that it aligns pretty nicely with the direction where we seem to think CMS is going and opening up and addressing some of those social determinants.”	6	National, 3-4 stars, >3 million members
Impact of services on star ratings	“As we think about doing something like that [introducing a new benefit], we really look to consider what might be the anticipated return on investment from something like that…. Then what could that mean financially to us as a health plan through the Medicare star program if we focus on specific measures and move from 4 to 4 and a half stars? That type of analysis routinely drives our dialogue with providers but also internally as to what it is we feel is the plan we want to invest in moving forward. Absolutely assess what value that brings as it relates to our star program.”	12	Regional, >4 stars, 100 000-250 000 members
Engaging with members	“We also are trying to be increasingly member centered…we do a lot of focus groups with patients, and we have patient advisors that will help tell us...what they feel like they need.”	13	Regional, >4 stars, <50 000 members
**Participants Highlighted Factors They Are Considering When Deciding Whether and How to Work With CBOs to Provide New Benefits**			
Ability of CBO to scale services	“A lot of the programs that we’re seeing that get amazing results focus on 100 to 200 families or individuals in a concentrated geographic neighborhood or city, and it’s a very high-touch model. But when thinking about how we scale that to 2 million people across 5 states with 5 different political systems, it tends to get disrupted.”	9	Regional, new,^b^ <50 000 members
Evidence of success	“The more evidence they [CBOs] have that their solution creates the outcomes to solve the problem that we have, the better. We’re more likely to move forward either with an intervention or potentially with a benefit around it. It’s proof points we’re interested in. If they’ve done previous pilots, we look at how rigorous their study designs are, whether a control group [was used or] not, and what type of outcomes are they looking at and over what period of time. We’re interested in those 3 categories that I mentioned. Quality of life, clinical outcomes, and more of the business financial result. So if they have that trifecta, that’s great. If they have 1 or 2 of them, then that’s okay, and we [kind of] make a case-by-case decision.”	6	National, 3-4 stars, >3 million members
Ability of CBO to scale services and deliver; desire to build on existing relationships	“Even with community-based organizations, there’s such variability in their ability to execute at a high level. They might be really, really good at doing meals in a very confined geography for a very specific population, but they’re not really able to take that to a higher level. So if they haven’t really demonstrated their ability to do that, we would be hesitant to put all our marbles in that box. So it is really multifactorial, and it really depends on what the service is and how close to the member it is, as opposed to is it really behind the walls and we’re really the face-to-face with the member, but we’re using those services. We would have to have service-level agreements about certain criteria that we would want met so that the members would not be negatively impacted if the administrative processes went awry. We’re responsible for all that.... So, a member can appeal any decision, and they can file grievances for anything. If they are not getting the services they believe they should, then they can file a grievance. If we’re not able to rectify that in a reasonable period of time, that is something that reflects us negatively. So we have a pretty high bar for making sure that whatever provider it is, is really going to be able to deliver.... Then we think about, ‘Do we already have providers who may have that skill or that ability, and is there a way to think differently about how we currently contract with them, as opposed to build[ing] something totally new?’ [Because] it’s very, very challenging to start out fresh. Because of data security and because of contracting, it can take 9 months to a year to get something like this up and going.... If we’re going to share data, it totally extends the time frame, because we have government contracts, so our data security is at the highest level.”	4	Regional, 3-4 stars, 50 000-100 000 members
Partnering with CBOs willing to share risk	“It’s interesting because it brings in new, when you think of operationalizing it [the addition of new services], that’s the biggest hurdle because it brings in a new provider type than we’re used to working with.... There’s an operational hurdle to figure out how that works.... I think it depends on the vendor...we love to work with vendors who are willing to take risks and have some skin in the game, but they don’t always exist for all these things.”	3	Regional, 3-4 stars, 100 000-250 000 members
**Additional Guidance From CMS Is Needed Before Participants Feel Comfortable Moving Forward With Addressing SDOH**			
Process of understanding the CHRONIC Care Act	“I think it’s a lot right now for our business side to digest. There’s clearly opportunities there, so it’s a matter of trying to seek out what those opportunities are and what makes sense. That’s kind of where we are, and still sort of the business area is digesting that, if that makes sense? I mean, I’m not sure there’s any one specific thing that they’re going to do in response to the CHRONIC Care Act, but it definitely puts more on your plate. You start looking at MA plans and what you can do and what you can’t do. There are a lot of rules, so that takes a lot of time, I think, for our compliance, our attorneys, to process, and to look, for our business folks to then look and say, ‘Are there opportunities here? If so, what are they?’”	11	Regional, >4 stars, 100 000-250 000 members
Policy evolution concerns	“That policy space is evolving as we speak.... So we’re all in kind of test-and-learn mode and probably will have a lot more to say about that over the next year or so. But it represents an evolutionary change, if not a revolutionary change, from the traditional approach within Medicare, whether it’s provisional fee-for-service Medicare or Medicare Advantage.... And doing this, starting off trying to do this in a social determinant space CMS is not necessarily sold on makes it doubly challenging. It’s unprecedented even to have something like food and security, or, rather, social determinants as coverable benefits on government plans. That’s one huge uphill battle to fight.”	6	National, 3-4 stars, >3 million members
How will CMS define SDOH?	“What is CMS really going to allow us to do and not do? The vague notion of social determinants of health has yet to be defined there. And we would hate to put our eggs in one basket and be moving down the road only to have CMS come back and redefine what they meant by that.”	9	Regional, new,^b^ <50 000 members
Moving forward cautiously	“There’s just a lot going on in this space, and I think one of the things that we’re trying to do is just be really cautious and tread lightly about—make sure we’re very thoughtful about how we go about adding services.”	2	National, 3-4 stars, >3 million members

Abbreviations: CBO, community-based organization; CHRONIC, Creating High-Quality Results and Outcomes Necessary to Improve Chronic; CMS, Centers for Medicare & Medicaid Services; COPD, chronic obstructive pulmonary disease; ROI, return on investment; SDOH, social determinants of health.

^a^ Organizations are identified by number for anonymity.

^b^ New plans are not eligible for a star rating until they have been active for at least 3 years.

#### Participants Highlighted Factors They Are Considering When Deciding Whether and How to Work With CBOs to Provide New Benefits

Participants discussed a number of decision points concerning whether and how to work with CBOs to provide services to address members’ SDOH. For plans looking to create a formal benefit and partner with CBOs to provide these services, some participants highlighted the importance of selecting a CBO partner that had the ability to scale services to a plan’s members; while plans may serve millions of members across states, CBOs frequently work on a significantly smaller scale. When selecting a partner, participants also expressed a desire to work with CBOs that had evidence of success and that are able to deliver (organization 4) in an effort to avoid members filing grievances and complaints that could affect the plan’s star rating. When deciding on whether and how to work with CBOs to provide benefits, some participants discussed the importance of partnering with CBOs that are willing to share the risk of providing a new benefit and building on existing relationships, given the challenges associated with developing new partnerships. Representative quotes are presented in Table 4.

#### Additional Guidance From CMS Is Needed Before Participants Feel Comfortable Moving Forward With Addressing SDOH

In discussing their decision-making efforts, participants from MA plans highlighted questions they still had about the CHRONIC Care Act at the time of the interview (following the passage of the CHRONIC Care Act but preceding the publication of the final rules) and how it would influence their decision-making. One concern mentioned by multiple participants was how CMS would define SDOH in the final regulations. Participants described recent policy changes as “an evolutionary change, if not a revolutionary change” (organization 6), which require that plans carefully consider how to respond. Given the challenges identified, participants were cautious about moving forward in developing benefits to address members’ SDOH. Representative quotes are presented in Table 4.

## Discussion

Interviews with representatives from 17 diverse MA plans revealed that plans recognize the influence of SDOH on the health status of their members. However, participants did not uniformly believe that it was the role of the health plan to directly address members’ SDOH-associated needs. Participants described 2 distinct methods of addressing members’ SDOH: through a supplemental benefit or by supporting and referring their members to CBOs. Participants described decision-making concerning how to address SDOH based on ROI, evidence of success, and the strength of CBO partnerships. Lastly, participants noted a need for further guidance from CMS on how the CHRONIC Care Act will be implemented.

Medicare Advantage plan representatives reported seeking to address SDOH either by introducing a formal benefit or by supporting and referring their members to community agencies. To our knowledge, there are no data from MA or other types of health care plans assessing outcomes related to different methods of addressing SDOH. As we heard from participants, there is limited evidence to guide plans in determining the best way to meet their members’ needs, including which, how, and for whom interventions work. Building this evidence base may be challenged by the lack of standardized data collection and evaluation measures.^30^ If plans cannot evaluate impact, then resulting cost savings, quality improvement, and member satisfaction will not be well understood. Having this information is important, as participants in this study noted these are the drivers of their decision-making. Therefore, tracking and reporting the success of program implementation and evaluation is important to expanding effective, evidence-based interventions. Additional research is needed to track MA plans’ decisions, ie, whether and how to offer a supplemental benefit to address SDOH, and to understand the impact of their decisions on members’ SDOH and associated health outcomes.

Restructuring a plan’s benefit package to include supplemental benefits comes with risk. Plans will not receive additional funding to provide supplemental benefits, and their medical loss ratios will not be adjusted to account for additional costs. In addition, plans offering benefits to address SDOH may attract members with greater social needs. As current MA risk-adjustment efforts are imperfect in accounting for social risk,^31,32^ plans that offer these benefits may be unfairly penalized. Thus, MA plans are considering ways to provide benefits that produce improved outcomes in specific populations. As noted by participants, this evidence base is crucial to decision-making concerning which benefits to provide.

Given the challenges that may be present for plans to expand benefits, it is not yet known how many plans will take advantage of the CHRONIC Care Act, as all expansions of benefits are optional. In 2019, CMS began to offer plans new flexibility in benefits.^33^ Early quantitative work on this expansion found very little uptake of new services, with expansions concentrated in large, established, health maintenance organization–style plans.^34^ In this study, we did not observe any patterns in responses associated with plan characteristic (ie, location, star rating, or size). However, it is possible that larger, more established plans have greater resources to contribute to the creation of supplemental benefits or working with CBOs or that they have already positioned themselves to address this increasing need before the CHRONIC Care Act was passed. It could also be the case that smaller, more nimble plans may be more amenable to engaging with their members directly in an effort to meet their SDOH-associated needs rather than source these activities out to CBOs. It is also possible that health maintenance organizations are better positioned than preferred provider organizations to address SDOH, given the capitated payment structure in health maintenance organizations. Future research should track new benefits through time to understand the response to the CHRONIC Care Act and how it varies by plan characteristics.

Centers for Medicare & Medicaid Services has begun to address some concerns about how to pay for these supplemental benefits in their 2020 call letter^35^; however, it remains to be seen what the benefits landscape will look like in 2020. To address the concerns of the participants interviewed in this study, CMS will need to balance granting flexibility with providing guidance so that plans feel confident in developing new initiatives without fear of audit, sanction, or loss of their contract with CMS. Given the investment that plans must make in determining which benefits to offer (with limited evidence) and plans’ hesitations to innovate in the midst of changing regulations, it is possible that the initial supplemental benefits addressing SDOH that are offered in 2020 will be modest.

To provide more benefits to address SDOH, MA plans may learn from the experience of other payment models in expanding their service offerings. While, to our knowledge, there is no evidence to date of how MA plans can better address SDOH, flexibility has existed to address SDOH and other patient needs in patient-centered medical homes,^36^ accountable care organizations (ACOs),^10,37,38,39,40,41^ and Medicaid Managed Care.^42,43,44^ In particular, ACOs may face many of the same challenges as MA plans as they begin to take on more capitated risk, ie, the need for a strong evidence base that addressing SDOH may improve patient outcomes and reduce costs, the ability to find partners who can provide these services, and clear guidance from CMS about what is permitted.^39^ In a 2014 qualitative study^38^ of ACO perspectives on SDOH, ACOs, similar to MA plans, were interested in expanding these types of benefits but did not always know how. The Hennepin model,^41^ in which a Medicaid Managed Care agency partners with a hospital, a community health center, and the county department of health to better coordinate services, is often seen as a successful example of how organizations can address SDOH through community partnerships. The past successes of Medicaid Managed Care in addressing SDOH may be an even more relevant example to MA plans on how an insurer can best address these concerns.^10^

### Limitations

Our study has limitations. Given the qualitative nature of our study and sample of plans, results may not be generalizable. We selected plans of varying sizes, geographic locations, and quality, and in total, these plans enrolled 13 million Medicare beneficiaries in 2018 (>65% of the MA market). However, participating plans may differ in their perspectives from nonparticipants, and therefore, these results are not intended to represent the universe of MA plans. For example, given our purposive and snowball sampling approach, plans with a particular interest in SDOH or innovative programming may have self-selected to participate. Nevertheless, our study is among the first to report findings from interviews with MA plan leaders, to our knowledge, and provides insights into leaders of MA plans’ decision-making as to how, as well as whether they should, address members’ SDOH in response to the CHRONIC Care Act.

## Conclusions

Findings from our interviews with participants from MA plans suggest that participants believe addressing SDOH is important. However, participants reported challenges in addressing members’ SDOH and apprehension about moving forward in this area without evidence and clear guidance from CMS. Therefore, it is likely that the introduction of new supplemental benefits in 2020 to address SDOH may be modest. Given the vulnerability of the population who may benefit from MA plans offering these expanded benefits, it is important that close attention be paid to how plans respond and the outcomes for Medicare beneficiaries and the US health care system more broadly.

## Notes

Supplement. **eAppendix.** Methods.
