# Supplementary material for: Perspectives of Medicare Advantage Plan Representatives on Addressing Social Determinants of Health in Response to the CHRONIC Care Act
Source: JAMA Netw Open. 2019 Jul 12;2(7):e196923. doi: 10.1001/jamanetworkopen.2019.6923 (PMC6628593; doi:10.1001/jamanetworkopen.2019.6923)
Supplement: Supplement. — eAppendix. Methods. [file jamanetwopen-2-e196923-s001.pdf]

Supplementary Online Content

Thomas KS, Durfey SNM, Gadbois EA, et al. Perspectives of Medicare Advantage plan representatives on addressing social determinants of health in response to the CHRONIC Care Act. *JAMA Netw Open*. 2019;2(7):e196923. doi:10.1001/jamanetworkopen.2019.6923

eAppendix. Methods

This supplementary material has been provided by the authors to give readers additional information about their work.

© 2019 Thomas KS et al. *JAMA Network Open*.

eAppendix. Methods

Domain 1: Research team and reflexivity
Personal characteristics

1. Which author/s conducted the interview or focus group? Interviews were conducted by E.A.G.
2. What were the researcher's credentials? E.g. PhD, MD. Interviewer E.A.G. has a PhD.
3. What was their occupation at the time of the study? Interviewer E.A.G. is a health services researcher and gerontologist.
4. Was the researcher male or female? Interviewer E.A.G. is female.
5. What experience or training did the researcher have? Interviewer E.A.G. has eight years of experience as a qualitative researcher, during which time she has conducted numerous interviews and focus groups with a variety of participants and in a range of settings.

Relationship with participants

6. Was a relationship established prior to study commencement? No, the interviewer did not have a prior relationship with interview participants.
7. What did the participants know about the researcher? e.g. personal goals, reasons for doing the research. Participants were informed about the study goals, the research institution, and the funding source. The interview guide was shared with participants prior to the interview in order to ensure that all appropriate potential participants were included in the interview.
8. What characteristics were reported about the interviewer/facilitator? e.g. Bias, assumptions, reasons and interests in the research topic. Participants were informed about the research institution and the funding source. The recruitment email read:
   I am an Investigator at Brown University and am working on a project in collaboration with Meals on Wheels America and funded by the Nonprofit Finance Fund. As part of this project, we are conducting interviews with Medicare Advantage plans to understand interest in addressing the population health of members, including social determinants of health, and receptivity to alternative payment models including Pay for Success.
   We recently spoke with another plan that shared your contact information with us. We are hoping that you, or someone in your organization, might be willing to participate in a one-time phone interview that should take less than one hour. During the interview we will ask questions about population health and innovative ways to improve health and decrease costs.
   Your involvement and details about your plan would be kept confidential. The ultimate goal is to use the data from these interviews to produce a report and potentially manuscripts that could shed light on the varied ways that MA plans are responding to the non-medical needs of their members and their thoughts about participation in Pay for Success initiatives.
   Would you be willing to talk with us? If you believe that you are not the right person within your organization, could you please refer us to someone who you think is more appropriate?
   Thank you, in advance, for your time.

Domain 2: Study design
Theoretical framework

9. What methodological orientation was stated to underpin the study? e.g. grounded theory, discourse analysis, ethnography, phenomenology, content analysis. The methodological approach to the study was content analysis.

Participant selection

10. How were participants selected? e.g. purposive, convenience, consecutive, snowball. Plans were selected through both purposive and snowball sampling. We first approached participants from plans we knew to be familiar with the topics of interest, and also asked all participants to identify participants from other plans that might be interested in taking part.
11. How were participants approached? e.g. face-to-face, telephone, mail, email. Potential participants were approached via email, using the recruitment script included in item 8, above.
12. How many participants were in the study? This study included 38 participants from 17 MA plans. Interviews were conducted with between 1 and 6 participants per plan.
13. How many people refused to participate or dropped out? Reasons? Due to the self-selecting nature of our recruitment efforts, we had no direct refusals. No plans or participants dropped out of participating.

Setting

14. Where was the data collected? e.g. home, clinic, workplace. Data were collected through a one-time semi-structured interview conducted via phone.
15. Was anyone else present besides the participants and researchers? No individuals were present except the participants and the researchers.
16. What are the important characteristics of the sample? e.g. demographic data, date. Because we were interested in decision-making, priorities, and perspectives at the plan level, we did not collect individual demographic data. We did, however, collect and present characteristics of participating plans (Table 1). These characteristics include whether the

© 2019 Thomas KS et al. *JAMA Network Open*.

plan has a national or regional focus, star rating, the absence or presence of a dual special needs plan, and enrollment.

Data collection

17. Were questions, prompts, guides provided by the authors? Was it pilot tested? The interview guide is included in this appendix. The interview guide was developed by the research team to attempt to understand perspectives of MA plans regarding addressing social determinants of health, and opinions around legislative and policy changes. It was reviewed among the project team and advisors, and piloted with one plan. It was then refined for clarify.
18. Were repeat interviews carried out? If yes, how many? Although no interviews were repeated with specific individuals, a repeat interview was carried out with the plan that participated in the pilot interview, although different individual participants were included.
19. Did the research use audio or visual recording to collect the data? Interviews were audio recorded with participants' consent.
20. Were field notes made during and/or after the interview or focus group? Field notes were taken by a member of the research team (J.B.) during and after each interview. The interviewer and PI reviewed field notes for completeness immediately after their completion.
21. What was the duration of the interviews or focus group? Each interview lasted about one hour.
22. Was data saturation discussed? The team continued to recruit participating plans until saturation was achieved, and no new information or themes were observed through additional interviews.
23. Were transcripts returned to participants for comment and/or correction? Transcripts were not returned to participants for comment and/or correction, although participants were encouraged to reach out to the research team with any questions or additional thoughts.

Domain 3: analysis and findings

Data analysis

24. How many data coders coded the data? Five researchers coded the data, working in rotating subteams of two or three members. Each researcher first individually coded each transcript, then met with their subteam members to reconcile the codes and create one master coded document for each transcript.
25. Did authors provide a description of the coding tree? The coding scheme is included in this appendix.
26. Were themes identified in advance or derived from the data? Themes were derived from the data.
27. What software, if applicable, was used to manage the data? NVivo version 12 was used to managed the data.
28. Did participants provide feedback on the findings? Participants did not provide feedback on the findings, although we plan to share all reports and papers that are produced using the data with participants upon publication.

Reporting

29. Were participant quotations presented to illustrate the themes/findings? Was each quotation identified? e.g. participant number. Yes, quotations were used and identified.
30. Was there consistency between the data presented and the findings? Yes. Representative quotes were included to provide support for all findings.
31. Were major themes clearly presented in the findings? Major themes were included as headings in the Results section, and also presented in tables with representative quotes.
32. Is there a description of diverse cases or discussion of minor themes? Yes. We were careful to appropriately represent the true range of nuanced perspectives.

© 2019 Thomas KS et al. *JAMA Network Open*.

Interview Guide

Thank you for agreeing to speak with us. We are interested in learning more about your plan's interest in addressing the health of your members. This includes social determinants of health. We're also interested in your receptivity to alternative payment models including Pay for Success.

Before we get started, I'd like to learn a little bit more about your role and background...

What is your current role? How long have you been in it?

What are your primary responsibilities?

Non-Medical Services

We understand that MA plans may be increasingly interested in providing non-medical services to improve the overall health and well-being of members. Oftentimes, this includes partnering with community-based, social service organizations to provide non- medical services to better manage the overall health of individuals with complex needs. What is your perspective on the value of community based, social services (such as transportation to appointments or home-delivered meals) as a component of your plan's design or services?

Relative to other aspects of benefit design, what priority do you place on providing non-medical services to improve the overall health of your members?

How have these priorities changed given CMS' new guidelines or the passage of the CHRONIC Care Act, which allows for more flexibility in covering non-medical benefits?

What characteristics describe your members with the greatest and most complex needs that would benefit from non-medical services? What do you see as the most needed/valuable services or benefits to address the needs of these members? Are these services currently provided?

If not, how do you decide what you cover? If not, how could these services be provided? What are the barriers to providing these services?

When you think about integrating new services, how receptive are your provider networks to these efforts?

Pay for Success/Outcomes-Based Financing

Now I want to switch gears a bit and talk about financing arrangements to pay for possible integration of these social services in plan benefit designs, specifically Pay for Success.

As you may know, In Pay for Success financing agreements, private investors provide upfront capital for the delivery of services, and these private investors are repaid if contractually agreed upon outcomes are achieved. In this arrangement, financial risk is shifted from service providers to investors, with investors underwriting the project based on the likelihood of pre-defined outcomes being achieved. Typically, an independent evaluator determines whether the agreed-upon outcomes have been met.

To what extent are you familiar with Pay for Success?

Has your plan discussed or thought about implementing Pay for Success initiatives to pilot solutions related to the non-medical needs of your members?

Is PFS an arrangement that would be attractive to your organization, perhaps in addressing social determinants of health among your members?

Why? Why Not?

If you aren't using Pay for Success, how else do you test or pilot innovative ideas for services or benefits? How do you decide what is worth testing? What informs your decisions?

For community-based organizations interested in partnering with you, how do you recommend they engage your organization?

What would you want to see from them in terms of evidence, business case, data readiness, HIPAA compliance, etc., in order to feel comfortable exploring a partnership?

What can an organization do to make themselves more appealing partners?

Concretely, what might that look like?

In Pay for Success, depending on the project you may need to engage legal counsel, finance, actuaries, compliance, quality, population health, and government affairs. How does, or would, your organization handle innovative projects that require the input and buy-in of so many parts of the organization?

Evaluation is critical to PFS. Under what circumstances would your organization be willing to share member utilization and cost data with project partners (potentially including independent validators and evaluators) in order to establish success baselines, and then to track project success metrics?

© 2019 Thomas KS et al. *JAMA Network Open*.

What are your current capabilities for evaluations of new programs and products? How might these capabilities play a role in working with a community-based organization and an independent evaluator?

What other thoughts, information, feedback would you like to share with us related to this work?

Do you have colleagues from other Medicare Advantage plans that you think would be willing to speak with us? Could you please share their contact information?

© 2019 Thomas KS et al. *JAMA Network Open*.

Coding Scheme

1. Background
    a. Interview participant attributes
    b. Organization attributes
    c. Mission (organizational or individual)
    d. Market competition
    e. Other
2. Non-medical services
    a. Perspective on non-medical services (broadly)
    b. Challenges/concerns with non-medical services
    c. Perspective on non-medical services as aspect of benefit design
    d. Characteristics of complex members who need non-medical services
    e. Services perceived to be important
    f. Services they offer
    g. Decision-making around service provision
    h. How services are provided
    i. Plan/provider interactions
    j. Other
3. CHRONIC/Changing CMS guidelines
    a. Regulatory environment of MA
    b. Familiarity with CHRONIC/changing CMS guidelines
    c. Impact of CHRONIC/CMS guidelines on interest in non-medical services
    d. Overall impact of CHRONIC/CMS guidelines
    e. Other
4. Members
    a. Description of members, member characteristics
    b. Plan interaction with members
    c. Efforts to appeal to members
    d. Other
5. Pay for Success awareness
    a. Overall familiarity with PFS
    b. Plan implementation of PFS
    c. Attractiveness of/receptivity to PFS
    d. Concerns, barriers, challenges
    e. Other
6. Organizational Operations
    a. If not PFS, how test innovative services or benefits?
    b. Inter-departmental relations
    c. Data sharing
    d. Data analysis capabilities
    e. Other
7. Community-based organizations
    a. Initiating the relationship
    b. Ongoing relationships
    c. CBO characteristics
    d. CBO concerns/challenges
    e. Other
8. Overall health care system
9. Good quote

© 2019 Thomas KS et al. *JAMA Network Open*.
